# Integrative analysis of multi-omics data reveals importance of collagen and the PI3K AKT signalling pathway in CAKUT

**DOI:** 10.1038/s41598-024-71721-8

**Published:** 2024-09-05

**Authors:** Jumamurat R. Bayjanov, Cenna Doornbos, Ozan Ozisik, Woosub Shin, Núria Queralt-Rosinach, Daphne Wijnbergen, Jean-Sébastien Saulnier-Blache, Joost P. Schanstra, Bénédicte Buffin-Meyer, Julie Klein, José M. Fernández, Rajaram Kaliyaperumal, Anaïs Baudot, Peter A. C. ’t Hoen, Friederike Ehrhart

**Affiliations:** 1 Department of Medical BioSciences, Radboud University Medical Centre, Nijmegen, The Netherlands; 2 Aix Marseille Univ, INSERM, MMG, Marseille, France; 3 Department of Bioinformatics-BiGCaT, NUTRIM/MHeNs, Maastricht University, Maastricht, The Netherlands; 4 Department of Human Genetics, Leiden University Medical Center, Leiden, The Netherlands; 5 Institut National de la Santé et de la Recherche Médicale (INSERM), U1297, Institute of Cardiovascular and Metabolic Disease, Toulouse, France; 6 Université Toulouse III Paul-Sabatier, Toulouse, France; 7 Barcelona Supercomputing Center (BSC), Barcelona, Spain; 8 CNRS, Marseille, France

**Keywords:** Systems biology, Nephrology, Kidney diseases

## Abstract

Congenital Anomalies of the Kidney and Urinary Tract (CAKUT) is the leading cause of childhood chronic kidney failure and a significant cause of chronic kidney disease in adults. Genetic and environmental factors are known to influence CAKUT development, but the currently known disease mechanism remains incomplete. Our goal is to identify affected pathways and networks in CAKUT, and thereby aid in getting a better understanding of its pathophysiology. With this goal, the miRNome, peptidome, and proteome of over 30 amniotic fluid samples of patients with non-severe CAKUT was compared to patients with severe CAKUT. These omics data sets were made findable, accessible, interoperable, and reusable (FAIR) to facilitate their integration with external data resources. Furthermore, we analysed and integrated the omics data sets using three different bioinformatics strategies: integrative analysis with mixOmics, joint dimensionality reduction and pathway analysis. The three bioinformatics analyses provided complementary features, but all pointed towards an important role for collagen in CAKUT development and the PI3K-AKT signalling pathway. Additionally, several key genes (CSF1, IGF2, ITGB1, and RAC1) and microRNAs were identified. We published the three analysis strategies as containerized workflows. These workflows can be applied to other FAIR data sets and help gaining knowledge on other rare diseases.

## Introduction

Congenital Anomalies of the Kidney and Urinary Tract (CAKUT) covers a wide range of structural malformations that result from defects in the morphogenesis of the kidney and/or urinary tract^1^. CAKUT affects three to six individuals per 1000 live births, constitutes the leading cause (~ 40%) of chronic kidney failure in childhood, and is a significant contributor to chronic kidney disease in adults^2,3^.

In recent years, alterations in more than 50 genes have been shown to be associated with CAKUT, but a clear genotype–phenotype relationship remains rare^3^. In addition, several lines of evidence suggest a clear environmental impact in the development of CAKUT, including significant associations between maternal diabetes, maternal obesity, low birthweight and CAKUT^4–6^. Therefore, better understanding of the pathogenetic mechanisms in CAKUT should involve analysis of biological markers such as gene expression data or proteomics which are closer to the genotype in order to capture the influence of both genetic and environmental impact. Multi-omics analysis and integration of this multilevel data should allow defining molecular pathways and networks connecting genotype and phenotype in CAKUT.

Therefore, the aim of our study was to improve on the understanding of CAKUT by integration of multi-omics data sets including miRNome, proteome and peptidome data obtained from amniotic fluid of pregnancies involving foetuses with CAKUT. In addition, all data was made findable, accessible, interoperable, and reusable (FAIR) in order to allow a seamless data exchange between international scientific groups. Similarly, the software tools created during the course of the EJP RD project will be dispatched and can be applied on other rare disease data sets.

## Results

**Fig. 1:**
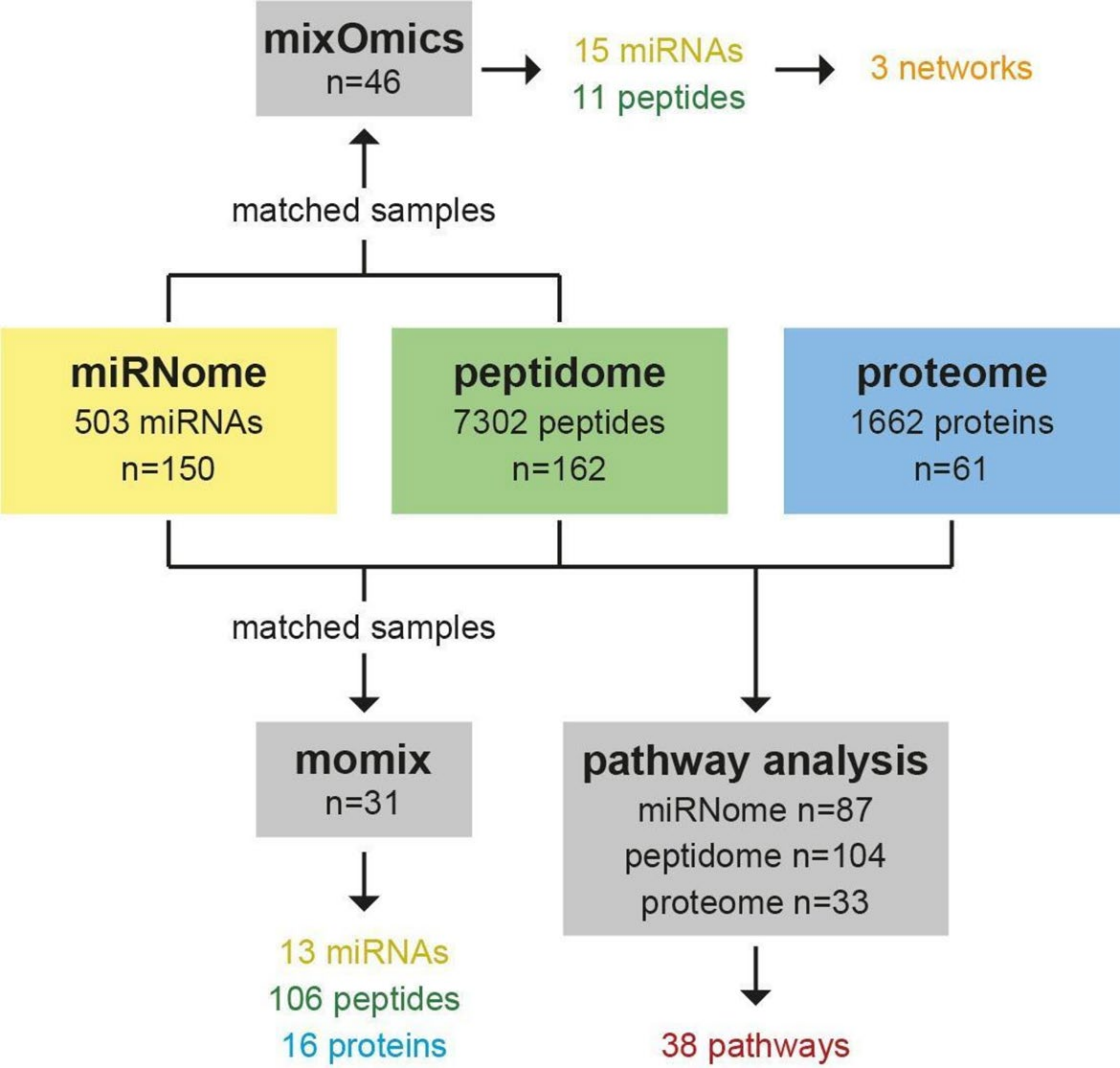
Analysis samples. Specification of the number of samples from each of the three omics data sets that was used for the three bioinformatics strategies (mixOmics, momix, and pathway analysis). For the mixOmics and the momix analysis, the number of samples was reduced, since these methods required samples from the same patient that matched between the different omics data sets. For the pathway analysis methods, all samples with sufficient clinical data were used for analysis. The results of each strategy are highlighted.

To increase our understanding of CAKUT disease aetiology, we performed multi-omics analyses on a total of 162 amniotic fluid samples. The omics types include previously published peptidome and proteome data from non-severe CAKUT and severe CAKUT patients, supplemented with novel miRNome data (see “Methods” section). We applied three different bioinformatics workflows to analyse and integrate this multi-omics data set. The workflows include intrinsic analysis using unsupervised (mixOmics) and supervised (momix) approaches, and extrinsic data analysis based on prior knowledge databases (pathway-level analysis). Each of the three complementary workflows used at least two types of omics data (Fig. 1). In order to facilitate data integration and analysis, both data and analysis scripts were FAIRified.

### FAIR data point creation

The multi-omics data sets were FAIRified by FAIR data experts within the European Joint Programme for Rare Diseases (EJP RD) following implementation choices and standards. Furthermore, a new catalogue was created in the EJP RD FAIR Data Point (FDP), which was supplemented with the CAKUT data set descriptions [https://w3id.org/ejp-rd/fairdatapoints/wp13/catalog/4cad6f79-a7e1-46ef-8706-37f942f4aaea]. This promotes reproducibility and reusability of the data in future analyses.

### Multi-omics integrative analysis with mixOmics

As a first approach to analyse the CAKUT multi-omics data, we used mixOmics, combining the miRNome and peptidome data with the mixOmics package^5^. This approach identifies common patterns among multiple omics datasets by projecting data into a small number of dimensions, where the number of dimensions or components can be specified. Only the samples that matched between the two omics data sets and were in the training cohort of the peptidome study^4^ were used (n = 46; 30 non-severe and 16 severe CAKUT cases). This was due to the nature of the analytic approach in the supervised classification method of the mixOmics package. In the mixOmics analysis, the proteomics data were not used, because there were a limited number of matching samples compared to the miRNome and peptidome data (Fig. 1).

**Fig. 2:**
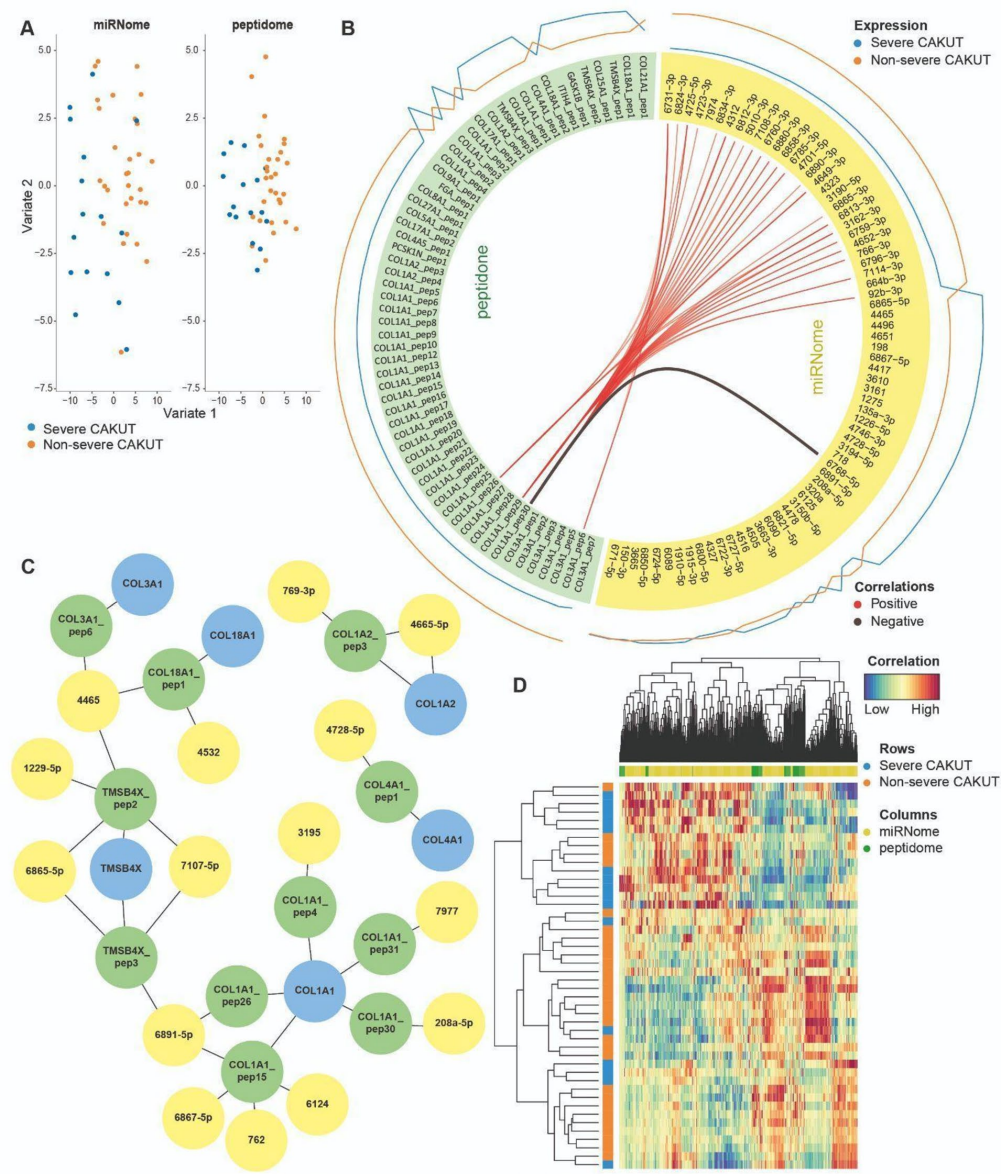
Integrative analysis of miRNome and peptidome data to identify combinations of variables from both omics data sets in comparison to the single-omics analysis using only peptidome data. (**A**) Multi-omics integration of miRNome and peptidome data using the block sPLS-DA method of the mixOmics package. Peptidome and miRNome data were matched by patient. Variates 1 and 2 indicate different latent components, where both peptidome and miRNome data are projected onto a smaller 5-dimensional subspace (see “Methods”). (**B**) Circos plot of correlations based on the sPLS-DA results using the miRNome (yellow) and peptidome (green) data of the first two components. miRNAs are indicated by their hsa-miR identifiers. Peptides are mapped to their respective proteins and multiple matches to the same protein are shown with the numbered suffixes. The exact peptide sequences can be found in the Supplemental Table 1. Only correlations scoring above 0.80 are shown. (**C**) Network-based integration of the miRNome, peptidome, and proteome data sets to depict the most relevant molecules identified by the mixOmics approach. The network is composed of the most relevant miRNAs (yellow) and peptides (green) based on sPLS-DA analysis as described in the “Methods” section. In this case, the peptide sequences were used to map peptides to proteins (blue) using sequence alignment (see “Methods”). Peptides and miRNAs are indicated as in (**B**). The larger network is a collagen and cytoskeleton network consisting of COL3A1, COL18A1, TMSB4X involved in cytoskeleton organisation, and COL1A1. The two smaller networks also include COL1A2 and COL4A1. (**D**) Unsupervised analysis between miRNAs and peptides displayed by a heatmap. The colours are based on their contributions to the first two components. Only miRNA and peptides with correlations above 0.80 are shown.

As part of the mixOmics analysis, Partial Least-Squares Discriminant Analysis (PLS-DA) and sparse PLS-DA (sPLS-DA) were used to identify a subset of variables that could explain the variability between non-severe CAKUT and severe CAKUT patients. It was noted that the peptidome data has a higher variance than miRNA data for the first two components in both PLS-DA and sPLS-DA analyses^6^ (Table 1 and Fig. 2A), which indicates that the peptidome data has a better segregation of non-severe CAKUT versus severe CAKUT patients than the miRNome. The main variability between the groups emerged from peptides that were derived from a variety of collagen proteins (Fig. 2B). These mainly include positive correlations for a large number of miRNAs with only three peptides (COL1A1_pep26, COL1A1_pep29, COL3A1_pep6). These observations confirmed the findings obtained using only the peptidome data^5^. Although classification accuracy is higher when just the peptidome data was used, multi-omics analysis revealed relationships between the miRNome and the peptidome (Fig. 2C). Only one negative relation was observed between the COL1A1_pep30 peptide and mir-hsa-6768-5p miRNA. For the highest scoring miRNAs and peptides of the mixOmics analysis, a network-based visualisation was performed. This network-based visualisation revealed a large collagen and cytoskeleton cluster (Fig. 2C) indicating major changes in the proteins, namely, TMSB4X, COL1A1, COL1A2, COL3A1, COL4A1, COL18A1. Furthermore, unsupervised analysis shown by heatmap clustering (Fig. 2D), confirmed strong correlations (> 0.8) between certain peptides and miRNAs (Supplementary Fig. 1). In conclusion, the mixOmics method highlights an important role of collagens on miRNome and peptidome level.

**Table 1:** Contribution scores per omics data for each of the two principal components of the principal component analysis, where sPLS-DA, a variable selection method was applied to select the optimal number of peptides and miRNAs.

Method	Component 1 (miRNA/peptidome)	Component 2 (miRNA/peptidome)
PLS-DA	80.47%/96.70%	80.77%/98.35%
Sparse PLS-DA	79.40%/96.43%	85.44%/96.15%

### Joint multi-omics dimensionality reduction analysis

**Fig. 3:**
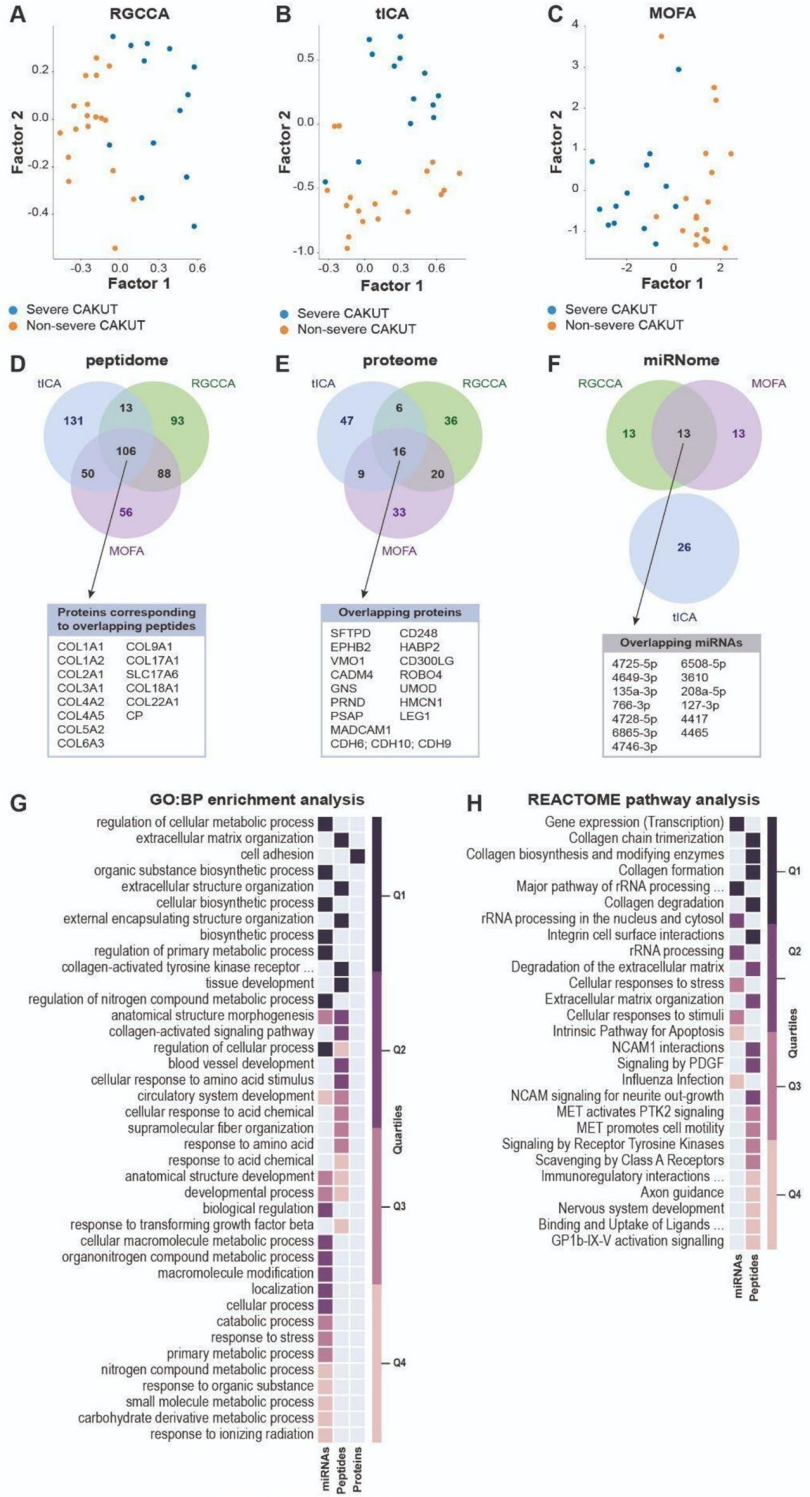
Joint multi-omics dimensionality reduction analysis. (**A**–**C**) Projections of all samples on the first two factors obtained by (a) RGCCA, (b) tICA and (c) MOFA. (**D**–**F**) Overlap of the top 5% peptides, proteins, and miRNAs selected by RGCCA, tICA, and MOFA analysis. (**G**) GO Biological Process enrichment analysis results of the features selected from different omics data by multiple methods. The significant results from different omics are filtered and integrated by orsum. The rank quartiles of the significant terms are coloured for the specific data sets. Enrichment scores can be found in Supplementary Table 5. (**H**) Reactome enrichment analysis results of the features selected from different omics data by multiple methods (there is no enrichment result for genes selected from the proteome data). The significant results from different omics are filtered and integrated by orsum. The colours indicate the quartile of the rank of the significant term for the specific dataset. Colours as in (**G**).

In the second strategy, we applied eight different unsupervised joint dimensionality reduction methods on the peptidome, proteome, and miRNome data using the momix notebook^7^. We used the 31 samples (18 non-severe CAKUT cases and 13 severe CAKUT cases) that matched between the three omics data sets. A joint dimensionality reduction method decomposes the omics datasets into omics-specific weight matrices and a joint factor matrix. We ran the dimensionality reduction methods to obtain the two most important factors (k = 2). Most non-severe and severe CAKUT patients could be separated by one of these two factors, which segregate the two groups (Fig. 3A–C). To evaluate the methods and choose the most relevant factor, we measured how well the two sample groups could be clustered. For each method and each factor, we used k-means clustering. We ran k-means 1000 times and counted the number of samples that were in the correct cluster in accordance with the clinical diagnosis. The baseline accuracy is 58% (18 over 31), which can be obtained by assigning all the samples to one of the two clusters. The accuracies of the joint dimensionality reduction methods range from 65 to 90% when from the two factors, the better segregating one is taken into account (Table 2).

**Table 2:** Accuracy of k-means clustering runs on each one of the two factors calculated by joint multi-omics dimensionality reduction methods. The bold numbers represent the higher accuracy obtained by each method.

Method	k-means 1st factor (%)	k-means 2nd factor (%)
RGCCA	**90**	58
tICA	65	**87**
MOFA	**87**	54
iCluster	**84**	62
intNMF	74	**81**
JIVE	**77**	74
MCIA	**77**	61
scikit-fusion	56	**65**

Based on the accuracy, we selected the three methods that were the most successful in separating non-severe and severe CAKUT patients, namely RGCCA, tICA, and MOFA (Fig. 3A–C). Within the weight matrices created by these methods, we used the weight vectors corresponding to the better of the two factors. We then used the absolute value of the weights assigned to the features and selected the top 5% of peptides, proteins, and miRNAs from each method for further analysis (Supplementary Tables 2–4).

We focused on the peptides and proteins identified as the top 5% by all three methods, and miRNAs identified by two methods, as there was no miRNA in common to all three methods (Fig. 3D–F). This resulted in 106 peptides, 16 proteins, and 13 miRNAs. These 106 peptides correspond to 15 proteins, mainly collagens: COL1A1, COL1A2, COL2A1, COL3A1, COL4A2, COL4A5, COL5A2, COL6A3, COL8A1, COL9A1, COL17A1, SLC17A6, COL18A1, COL22A1, and CP. None of these 15 proteins were identified in the top 5% of the proteome, however, some corresponded to related proteins. For instance, Cadherins (CDH6, CDH9, CDH109) and CADM4 play a role in calcium-dependent cell adhesion. Furthermore, ROBO4, UMOD, HABP2, MADCAM1, HMCN1, and EPHB2 have been indicated to be involved in cell adhesion, cell junctions and/ or the migration of one or more specific cell types. Overall, this indicates that the peptidome and the proteome identify different proteins but similar processes.

We performed enrichment analysis to identify the most important biological processes associated with the selected peptides, proteins, and miRNAs (Supplementary Tables 2–4). In this analysis, we used the proteins selected from the proteome data, the proteins corresponding to the selected peptides and the genes targeted by the selected miRNAs. We used orsum^8^ in order to present the enrichment results and to filter redundant annotation terms (Fig. 3G). Five Gene Ontology Biological Process (GO-BP) terms are significantly enriched in both the miRNome and peptidome data, mainly indicating misregulation of organ structure and development in non-severe CAKUT patients versus severe CAKUT patients. “Cell Adhesion” (GO:0007155) is the only significantly enriched GO-BP term in the proteomics data. Proteins corresponding to the selected peptides are further enriched in extracellular processes, including the process entitled “collagen-activated tyrosine kinase receptor signaling pathway” (GO:0038063). Cell adhesion and collagen related pathways are also significant when REACTOME pathways are used in the enrichment analysis (Fig. 3H). Finally, for the miRNome data, the REACTOME enrichment analysis of the genes targeted by the selected miRNAs mainly revealed rRNA and transcription processes. The GO-BP enrichment analyses indicated a role for the miRNA regulated genes in metabolomics and biosynthesis, for which misregulation could affect organ structure and development.

### Pathway-level analysis

**Fig. 4:**
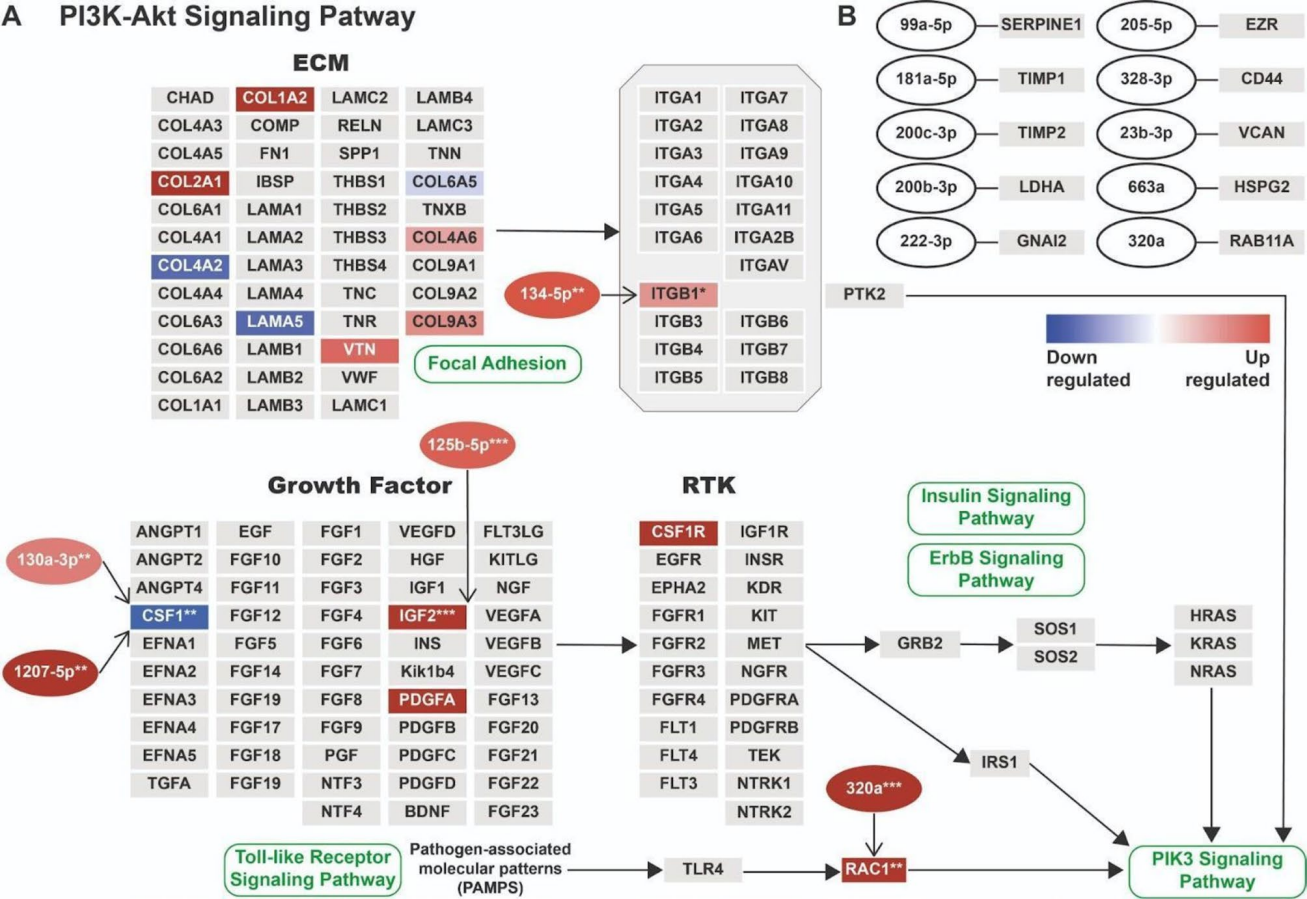
Pathway enrichment analysis. Visualisation of the interacting differentially expressed proteins/peptides/miRNAs in the WikiPathways pathway database on the combined miRnome, peptidome, and proteome data. Rectangular nodes represent protein products as determined from the peptidome and proteome, ellipses represent miRNAs indicated by their hsa-miR identifiers. (**A**) Visualisation of the PI3K-Akt Signalling Pathway as adjusted from WikiPathways (WikiPathways:WP4172). Only a part of the pathway is shown from the larger pathway to emphasise the section where most differential expressions occurred. Blue indicates downregulation and red upregulation, as indicated by the gradient bar. Asterisks indicate the enrichment significance (p-value). Grey nodes mean that there was no expression data found. On the one hand, we found that IGF2, ITGB1, and RAC1 were upregulated in the same direction as their miRNAs. On the other hand, CSF1 was downregulated in contrast to its targeting miRNAs, which were both upregulated. (**B**) The 10 remaining significantly linked miRNA and proteins, from the 15 interactions that were identified in total. The gene products were selected only when either peptidome or proteome indicated significant levels of differential regulation, as well as, the significant miRNAs targeting them.

We analysed the CAKUT omics data for overrepresented pathways within the WikiPathways database^9^. From 634 pathways in the database, 38 pathways were overrepresented and had a link between miRNA and protein (or peptide mapped into protein) based on the CAKUT patient data. In these pathways, we found 15 links between miRNome and proteome where both interaction partners are significantly differentially expressed. The “PI3K-Akt Signalling Pathway” (WikiPathways: WP4172)^10^ is a major regulator of the cell cycle and it contained five links between miRNAs and the peptidome or proteome (Fig. 4A). The 10 remaining links between miRNA and proteins are indicated in Fig. 4B.

The PI3K-Akt pathway also includes certain collagens that had been associated with CAKUT in the original study^4^. However, we could not identify any significant links between these proteins and the miRNome. Instead, in this pathway, we found significant links between four gene products (CSF1, IGF2, ITGB1, and RAC1) and five miRNAs (hsa-miR-130a-3p, hsa-miR-1207-5p, hsa-miR-125b-5p, hsa-miR-134-5p, and hsa-miR-320a). A significant link indicates that a differentially expressed miRNA binds to the mRNA of a differentially expressed protein indicating a regulatory connection.

## Discussion

Our main result was that we identified affected pathways and networks in CAKUT, and thereby aid in getting a better understanding of its pathophysiology. We did this by re-using existing data, combining it with new data (miRNome) and using available data from knowledge bases for analysis as a strategy to overcome the notorious shortage of data for rare diseases. The three bioinformatics analyses pointed towards an important role for collagen in CAKUT development and the PI3K-AKT signalling pathway. Additionally, several key genes (CSF1, IGF2, ITGB1, and RAC1) and microRNAs were identified. Finally, driven by the EJP RD project, we applied open science and the FAIR principles to multi-omics rare disease data sets to facilitate their integration and analysis with relevant external data resources and support their reusability by the scientific community for (rare) disease research.

Using the output from the mixOmics approach a network was identified related to collagen and cytoskeleton remodelling, consisting of COL3A1, COL18A1, TMSB4X, and COL1A1, and two smaller networks including COL1A2 and COL4A1. COL3A1, COL18A1, COL1A1, COL1A2 and COL4A1 are collagens and TMSB4X is a G-actin binding protein involved in cytoskeleton formation. In detail, COL3A1 is involved in blood vessel formation and if mutated can cause a vascular type of Ehlers-Danlos syndrome^11^. COL4A1 is also involved in angiogenesis and if mutated can cause several types of hereditary angiopathies. In a study from Plaisier et al.^12^ basement membrane defects in kidney and skin were detected in patients with mutations in COL4A1. Animal models typically express defects in blood vessel stability resulting frequently in perinatal cerebral hemorrhage but also eye and kidney malformations^13^. COL18A1 is involved in Knobloch syndrome 1, which is characterised by malformations of the eye and glaucoma^14^. There are several studies on animal models available, which report abnormal eye, head and heart formation and one study reported also abnormal kidney filtration capacity in their mouse model^15^. COL1A1 can cause several forms of osteogenesis imperfecta, variations of Ehler-Danlos syndrome and other bone mineral density variation disorders^16^. Mouse models exist, their phenotype is characterised by high occurrence of bone fractures^17^. COL1A2 can also cause several forms of osteogenesis imperfecta but also the cardiac valvular type of Ehler-Danlos syndrome^18^. Neither COL1A1 nor COL1A2 has been linked to renal abnormalities before. For TMSB4X there are no clear links to diseases known. As a G-actin binding protein involved in cytoskeleton formation and maintenance it was in vitro shown to be essential for coronary vessel development and cell migration^19^.

In this analysis, we used a supervised classification approach with the mixOmics method of the mixOmics package, which requires matching samples among omics data sets. Since the number of overlapping samples in all sets decreased when the proteomics data were included in the mixOmics-based analysis, we decided to exclude the proteomics data for this specific analysis. MixOmics proposes two approaches, sPLD-DA and PLD-DA. The difference between sPLS-DA and PLS-DA was insignificant, probably because sPLS-DA is expected to be beneficial over PLS-DA for high dimensional data^20^. Additionally, mixOmics analysis allowed the identification of a collagen-related cluster solely based on the peptidome and miRNome data. The main variance in the data stemmed from a range of miRNAs that could be connected to a small number of peptides (Fig. 2B). Most of these relations were positive correlations, while only hsa-miR-6768-5p and COL1A1_pep30 (ADGQpGAKGEpGDAGAKGDAGPpGP) had a negative correlation. hsa-miR-6768-5p has not been previously identified or predicted to affect COL1A1. While an important role for collagen in CAKUT was previously established^4,12,21^, COL1A1 has not specifically been linked to CAKUT. Furthermore, this work highlights potential novel miRNA and peptide relations, which might be relevant to study in order to get a better understanding of CAKUT.

Unsupervised joint dimensionality reduction analysis with the momix notebook identified the most relevant molecules from the three omics data sets. We further selected the results of the three best performing joint dimensionality reduction methods among the eight tested methods. From the proteome analysis, CDH6 (P55285), CDH9 (Q9ULB4), and CDH10 (Q9Y6N8) are particularly interesting, as these cadherins regulate hippo signalling, which plays a role in kidney and urinary tract development (Fig. 3E)^22,23^. Furthermore, UMOD (P07911) was previously associated with medullary cystic kidney disease, familial juvenile hyperuricemic nephropathy, and glomerulocystic kidney disease^24^. Whether mutations in UMOD are a cause of CAKUT is still under debate^24^. The peptide analysis revealed COL4A5 (P29400) as an interesting protein, as it is one of the glomerular basement membrane proteins that cause Alport syndrome^25^. COL4A1 (P02462) is also of interest. This protein is identified by all the three best performing methods due to different peptides (MOFA and RGCCA found the peptide COL4A1_pep1, tICA found the peptide COL4A1_pep2) (Supplemental Table 1) and it is associated with kidney diseases^12,21,26^. Among the enriched annotation terms, cell–cell adhesion and extracellular matrix organization are known to play a role in the ureteric bud branching^27^.

Comparing the momix and mixOmics workflows, there is an overlap in the identified molecules of interest, including COL1A1, COL1A2, COL3A1, and COL18A1. In the GO enrichment analysis, we obtained different annotation terms indicating that momix and mixOmics approaches are complementary.

The analysis at pathway-level used the molecular interactions of WikiPathways, a pathway database extended with miRNA-target information as a backbone to investigate the interactions of interest. The advantage of this method is that it integrates prior knowledge into the analysis, which is especially important when the signal extracted from the data is low. Using this pathway analysis method, we identified 15 functional links between significant differentially expressed proteins and the miRNome. The PI3K-AKT signalling pathway hosts five of these interactions between the different omics data sets, making this the most relevant pathway for CAKUT disease progression (Fig. 4A). In addition, it harbours several collagen proteins previously identified by the other methods as well. Involvement of the PI3KT-AKT pathway showed up in a study on transcriptomics data of CAKUT patients^28^ and via the MDM2 gene on another study using miRNA data^29^. Collagen modifications have been associated with the development of CAKUT^4,21,30^. Whether collagens are causally involved remains to be determined. Kitzler et al., described that COL4A1 variants could be a potential novel cause of autosomal dominant CAKUT in humans leading predominantly to a vesicoureteral reflux and isolated (nonsyndromic) CAKUT phenotype^21^. Variants in different extracellular matrix proteins or proteins that interact with the ECM have been described^30^. Collagens make up a large part of the ECM and remodelling of the ECM, potentially due to such variants, are likely reflected by changes observed in collagen fragments in amniotic fluid. In addition, it is likely that the increased abundance of collagen fragments in amniotic fluid represents ECM remodelling due to kidneys with dys-/hypoplasia, cysts and hyperechogenicity even without gene variants that specifically target the ECM (e.g. *HNF1B* variants)^4^.

The other interactions from i.a. “Focal Adhesion” (WikiPathways: WP306) or “Senescence and Autophagy” (WikiPathways: WP28806) pathway, are shown in Fig. 4B. The limitation of the pathway-level analysis is the dependence on knowledge databases of molecular interactions. Nonetheless, for both pathways and miRNA-target interactions, there are several options regarding analysis. On the one hand, WikiPathways is an open, community created, and expert curated database^9^. The contributions that define the content are dependent on published literature, and the pathways undergo regular curation to be updated with current findings. On the other hand, miRTarBase is a miRNA-target interaction database that provides manually selected, experimentally validated miRNA-target interactions from published literature^31^. Integrating analysis methods using these and other databases to cross validate the information measured on patient material is important to draw relevant conclusions for disease research.

Altogether, the different bioinformatics strategies and methods presented in this study offer a complementary spectrum of possible multi-omics strategies, which can be used for the analysis of rare disease data sets. Notably, most of these methods identified the same (functional) group of genes, with differences on the weighing of correlation statistics or the use of prior knowledge supported methods. Importantly, methods based on mathematical analysis, ignoring existing biomedical knowledge, allow us to identify potentially interesting findings in a hypothesis free manner. Pathways, or approaches based on prior knowledge in general allow us to select functional and molecular interactions from the given data to support a biomedical interpretation of the results. We demonstrated that a combination of these strategies is advantageous for the analysis of (multi-)omics data in the field of rare diseases.

There is an increasing demand towards open science, which requires providing the data, analysis tools, and whole workflows FAIRly available together with the results. This significantly increases the possibility to reproduce results and counteract the current crisis in reproducibility and trust in scientific studies. This demand is especially high in the rare disease field where the naturally limited number of patients, samples, and data has ever since encouraged international and interdisciplinary collaborations to pool data and exchange methods in how to deal with low sample numbers. To this purpose, we hope to aid research reliability and reproducibility by providing both FAIR metadata and workflows as presented in this study and supported by the EJP RD.

In summary, we provided several different complementary bioinformatics strategies and their results that, in combination, could identify biologically relevant biological molecules, pathways, and networks from multi-omics rare disease data sets both in an unsupervised and supervised manner. The identified proteins, peptides, and miRNAs highlight modules relevant for CAKUT disease and they can be used for future investigations and experimental validation. Finally, the application of open science and FAIR principles in this study contributes to the transparency and reusability of data and workflows in, but not limited to, the rare disease field.

## Methods

### Multi-omics data sets

The CAKUT multi-omics data set was obtained from a previously published study and reinvestigated in collaboration with the authors of the original study^4,32^. The ethical approval was given by the patient protection committee of the French south-west and overseas-1 departments (approval number DC-2016-2611). The initial study contains amniotic fluid samples from proteome and peptidome. Here we added novel miRNome data from amniotic fluid samples, which were derived from the same patients as described below. As stated in the previously published studies, the study protocol was approved by the national ethics committees (France, RCB 2010-AO1151-38; Belgium S 55406 and B32220096569), and informed consent was obtained from all participants. All experiments were performed in accordance with relevant named guidelines and regulations. In total 162 individuals were studied, of which 104 samples had a clear postnatal outcome. Patients were diagnosed with either non-severe CAKUT, that were patients with a normal GFR (glomerular filtration rate), moderately reduced GFR (60–90 ml/min per 1.73 m^2^) or reduced GFR (< 60 ml/min per 1.73 m^2^) at two years of age, or severe CAKUT, that patients were diagnosed postnatally with severe renal failure, chronic kidney failure or the renal phenotype that lead to a termination of pregnancy.

In total, the abundances of 7302 peptides were measured in the amniotic fluid samples of 162 subjects, 503 miRNAs were significantly detected from 150 samples and 1662 proteins were detected in 61 samples. Each of these three omics data sets includes non-severe CAKUT vs. severe CAKUT cases.

### miRNome sample collection and analysis

For miRNA analysis, amniotic fluid samples were collected in a prospective multicenter observational study focusing on foetal bilateral CAKUT as part of a clinical trial (https://clinicaltrials.gov/ct2/show/NCT02675686). The CAKUT disease severity was defined based on the renal status after two years of postnatal clinical follow-up.

The total RNA was isolated using the Agilent RNA 6000 Pico kit protocol (5067-1513) and microRNAs were profiled using Agilent microRNA slides (Sanger miRBase release 21). The samples were labelled and hybridised according to the Agilent's microRNA Complete Labeling and Hybridization Kit protocol (5190-0456), followed by Spike-ins with the Agilent's microRNA Spike-In Kit protocol (5190-1934) and analysed using Agilent's High-Resolution Microarray Scanner GS2505_C. Features were called using the Agilent Feature Extraction software (version 11.0.1.1) and sample intensities were normalised using quantile normalisation (RMA). The miRNome data is available at 10.5281/zenodo.7866785.

### FAIR data point and data deposition

We used a FAIR Data Point (FDP) to describe the CAKUT multi-omics data set. The FDP is a metadata service that provides descriptions about resources^33^. It uses Data Catalogue Vocabulary (DCAT) to capture the resources metadata. The FDP serves descriptions of resources to both humans and machines, which makes integration of different data sets easier and allows reproducing results from previous studies. The human users who visit the FDP see the resource descriptions as HTML documents and the machine gets semantic resource descriptions as an RDF document. We used the FDP [https://w3id.org/ejp-rd/fairdatapoints/wp13] created for the EJP RD project to describe the CAKUT multi-omics data sets.

### Workflow specifications

Workflows from each of the three different types of analyses have been registered at the WorkflowHub [https://workflowhub.eu/documents/8?version=1], a scientific FAIR workflow registry. From this registry, each workflow can be downloaded together with all required scripts and data files as a single package^34^. This facilitates re-analyses of the same data sets but also application of the workflows to additional data sets.

### Multi-omics integrative analysis with mixOmics

We used the mixOmics package^35^ (version 6.10.9) with PLS-DA (Partial Least Squares Discriminant Analysis Discriminant Analysis) and sPLS-DA (sparse PLS-DA) supervised classification. The sPLS-DA method allowed for variable selection on each omics. As in principal component analysis, sPLS-DA projects large input data into a smaller dimensional space, with each component representing a different dimension. From the peptidome data, 53 samples were used for training and 51 samples were used for validation. The samples were assigned to each of the groups in the initial study^4^. In line with this grouping, of the samples that matched between the peptidome and miRNome, 41 were used for training and 46 for validation. Nonetheless, when matching samples of the proteome to the other two omics data sets, there were only 23 and 10 samples for training and validation remaining respectively. Thus, the proteome data was not used in the mixOmics analysis. For the sPLS-DA classification, a maximum of five components were chosen, where each component represents a separate dimensional subspace for data projection. The first component uses 50 miRNAs and peptides, the second component uses 20 miRNAs and 10 peptides, whereas all other three components use all miRNAs and peptides. Peptides are mapped to their respective proteins and multiple matches to the same protein are shown with the numbered suffixes. The exact peptide sequences can be found in the Supplemental Table 1.

For the network shown in Fig. 2C: (1) the edges between miRNAs and peptides are determined by their statistical significance, which was based on the multi-omics analysis of the training data. (2) The edges between miRNAs and proteins are identified using the known miRNA–mRNA biological links provided by the mirTarBase database, version 8.0^31^. (3) The edges between proteins and peptides are identified by aligning peptide sequences against protein sequences using NCBI BLAST.

We used R version 4.0.3 for the analysis. All R packages necessary to run these scripts are specified in the Docker file included at [https://workflowhub.eu/documents/8?version=1].

### Multi-omics integrative analysis with joint dimensionality reduction using momix

We applied eight joint dimensionality reduction methods on peptidome, proteome, and miRNome data for 31 samples (18 non-severe CAKUT cases and 13 severe CAKUT cases) matched in the different omics data sets. For this, we used the momix notebook^7^. The methods include: iCluster^36^, Integrative NMF (intNMF)^37^, Joint and Individual Variation Explained (JIVE)^38^, Multiple Co-Inertia Analysis (MCIA)^39^, Multi-Omics Factor Analysis (MOFA)^40^, Regularized Generalized Canonical Correlation Analysis (RGCCA)^41^, matrix-tri-factorization (scikit-fusion)^42^, and tensorial Independent Component Analysis (tICA)^43^.

We used these methods to obtain two factors in the reduced dimensional space. We observed that non-severe CAKUT and severe CAKUT patients could be separated by one of the two factors (Fig. 3A–C). We used k-means clustering to select the factor, which better segregated non-severe CAKUT and severe CAKUT patients. We ran 1000 k-means clustering cycles on each factor independently, and calculated the accuracy. The two labels are assigned randomly to the two clusters, the accuracy is measured; then labels are switched, the accuracy is measured again; the factor with the highest accuracy is considered the best. Based on these accuracies, we also selected the best performing three methods. The top three methods, RGCCA, tICA, and MOFA, obtained 87% or 90% accuracy using a single factor (Table 2). We used the results from these methods for subsequent analyses.

From the weight matrices created by the top three methods, we used the weight vectors corresponding to the selected better segregating factor. Using the absolute value of the weights assigned to the features, we selected the top 5% peptides, proteins and miRNAs. Among these top 5% molecules identified by the top three methods, we focused on the peptides and proteins identified by all three methods, and the miRNAs identified by two methods (RGCCA and MOFA), as there was no miRNA in common to all three methods.

For robustness, we used the top features identified by multiple methods. This resulted in 106 peptides, 18 proteins, and 13 miRNAs (note that for proteins, 16 features are selected but one feature is "P55285; Q9Y6N8; Q9ULB4", which corresponds to three cadherins, CHD6, CHD9 and CHD10). The peptides were mapped to proteins/genes using the UniProt Retrieve/ID mapping module (https://www.uniprot.org/uploadlists/)^44^. For miRNA to target gene mapping, we used hsa_MTI.xlsx from miRTarBase (Release 8.0)^31^.

The enrichment analysis was performed using g:Profiler^45^. In the enrichment analysis, we continued only with CDH6 from the cadherins that were measured jointly in proteomics analysis (P55285; Q9Y6N8; Q9ULB4), to prevent inflation in the enrichment results. For the presentation and the filtering of redundant annotation terms in the enrichment results, we used orsum^8^.

### Pathway-level analysis to detect functional links

The peptidome and the proteome data sets were quantile normalised and log2 transformed as previously described^46,47^. Before transformation, peptide IDs were mapped to protein IDs, and summarised into single protein-level values using geometric mean^32^. The miRNome data set was already normalised and transformed, thus the information of their target genes could be added to each miRNA ID without additional data manipulation, using the information provided by miTaRBase. As a result, all three data sets had been mapped to their appropriate gene product-level identifiers.

Once the data sets were prepared, we applied one-predictor logistic regression for each protein- or miRNA-level and obtained the effect size (log2 fold change) and p-values. Each element of each data set (miRNA/peptide/protein) was deemed significantly differentially expressed if the corresponding p-value was less than 0.05.

In this analysis, we created an extended pathway network, using the WikiPathways repository (Version 20210110). For the pathway-level analysis, first each of the three omics data sets was analysed to identify overrepresented pathways. Subsequently, pathways associated with the significant miRNA-protein links were detected. A miRNA-protein link may possibly be implying causality, if both a miRNA and its target are differentially expressed.

Pathways, which are overrepresented and contain at least one link from a significant miRNA either to a significant peptide or to a significant protein were identified. More specifically, a pathway was selected if it meets two conditions: (1) a gene product in the pathway was significantly differentially expressed by either the peptidome or proteome, or (2) there exists a miRNA, which targets the gene product, and the miRNA is significantly differentially expressed.

Finally, since the selected pathways only included information of gene products, they were extended using the miRNA targeting information when necessary. A visualisation of the selected pathway with study data and additional information was created.

## Supplementary Information

Supplementary Table 1.

Supplementary Table 2.

Supplementary Table 3.

Supplementary Table 4.

Supplementary Table 5.

Supplementary Figure 1.

## Data Availability

The CAKUT multi-omics data sets for the proteomics and peptidomics data sets are available with the original studies^5,22^ and the repositories mentioned respectively. The proteomics data is on ProteomeXchange (PXD022926), peptidome data is available also here: 10.5281/zenodo.10497903 and the miRNome data is available at 10.5281/zenodo.7866785. We added a FAIR data point to describe the CAKUT data sets: https://w3id.org/ejp-rd/fairdatapoints/wp13/catalog/4cad6f79-a7e1-46ef-8706-37f942f4aaea. All data analysis code is available at the Workflowhub registry: https://workflowhub.eu/documents/8?version=1.

## References

[1] Vivante, A., Kohl, S., Hwang, D. Y., Dworschak, G. C. & Hildebrandt, F. Single-gene causes of congenital anomalies of the kidney and urinary tract (CAKUT) in humans. *Pediatr. Nephrol.* **29**, 695–704. 10.1007/s00467-013-2684-4 (2014). 24398540 10.1007/s00467-013-2684-4 PMC4676405

[2] Nicolaou, N., Renkema, K. Y., Bongers, E. M., Giles, R. H. & Knoers, N. V. Genetic, environmental, and epigenetic factors involved in CAKUT. *Nat. Rev. Nephrol.* **11**, 720–731. 10.1038/nrneph.2015.140 (2015). 26281895 10.1038/nrneph.2015.140

[3] Knoers, N. & Renkema, K. Y. The genomic landscape of CAKUT; you gain some, you lose some. *Kidney Int.* **96**, 267–269. 10.1016/j.kint.2019.03.017 (2019). 31331462 10.1016/j.kint.2019.03.017

[4] Klein, J. *et al.* Amniotic fluid peptides predict postnatal kidney survival in developmental kidney disease. *Kidney Int.* **99**, 737–749. 10.1016/j.kint.2020.06.043 (2021). 32750455 10.1016/j.kint.2020.06.043

[5] Rohart, F., Gautier, B., Singh, A. & Le Cao, K. A. mixOmics: An R package for ’omics feature selection and multiple data integration. *PLoS Comput. Biol.* **13**, e1005752. 10.1371/journal.pcbi.1005752 (2017). 29099853 10.1371/journal.pcbi.1005752 PMC5687754

[6] Le Cao, K. A., Boitard, S. & Besse, P. Sparse PLS discriminant analysis: Biologically relevant feature selection and graphical displays for multiclass problems. *BMC Bioinform.* **12**, 253. 10.1186/1471-2105-12-253 (2011). 10.1186/1471-2105-12-253 PMC3133555 21693065

[7] Cantini, L. *et al.* Benchmarking joint multi-omics dimensionality reduction approaches for the study of cancer. *Nat. Commun.* **12**, 124. 10.1038/s41467-020-20430-7 (2021). 33402734 10.1038/s41467-020-20430-7 PMC7785750

[8] Ozisik, O., Terezol, M. & Baudot, A. orsum: A Python package for filtering and comparing enrichment analyses using a simple principle. *BMC Bioinform.* **23**, 293. 10.1186/s12859-022-04828-2 (2022). 10.1186/s12859-022-04828-2 PMC9308244 35870894

[9] Martens, M. *et al.* WikiPathways: Connecting communities. *Nucleic Acids Res.* **49**, D613–D621. 10.1093/nar/gkaa1024 (2021). 33211851 10.1093/nar/gkaa1024 PMC7779061

[10] Hanspers, K., Riutta, A., Willighagen, E. L. & Weitz, E. *PI3K-Akt signaling pathway (Homo sapiens)*. http://wikipathways.org/instance/WP4172 (2022).

[11] Schwarze, U., Goldstein, J. A. & Byers, P. H. Splicing defects in the COL3A1 gene: Marked preference for 5’ (donor) spice-site mutations in patients with exon-skipping mutations and Ehlers-Danlos syndrome type IV. *Am. J. Hum. Genet.* **61**, 1276–1286. 10.1086/301641 (1997). 9399899 10.1086/301641 PMC1716081

[12] Plaisier, E. *et al.* COL4A1 mutations and hereditary angiopathy, nephropathy, aneurysms, and muscle cramps. *N. Engl. J. Med.* **357**, 2687–2695. 10.1056/NEJMoa071906 (2007). 18160688 10.1056/NEJMoa071906

[13] Gould, D. B. *et al.* Mutations in Col4a1 cause perinatal cerebral hemorrhage and porencephaly. *Science* **308**, 1167–1171. 10.1126/science.1109418 (2005). 15905400 10.1126/science.1109418

[14] Sertie, A. L. *et al.* Collagen XVIII, containing an endogenous inhibitor of angiogenesis and tumor growth, plays a critical role in the maintenance of retinal structure and in neural tube closure (Knobloch syndrome). *Hum. Mol. Genet.* **9**, 2051–2058. 10.1093/hmg/9.13.2051 (2000). 10942434 10.1093/hmg/9.13.2051

[15] Utriainen, A. *et al.* Structurally altered basement membranes and hydrocephalus in a type XVIII collagen deficient mouse line. *Hum. Mol. Genet.* **13**, 2089–2099. 10.1093/hmg/ddh213 (2004). 15254016 10.1093/hmg/ddh213

[16] Byers, P. H. in *Connective Tissue and Its Heritable Disorders: Molecular, Genetic, and Medical Aspects.* (ed P. M.; Steinmann Royce, B.) 317–350 (Wiley-Liss, 1993).

[17] Chen, F. *et al.* First mouse model for combined osteogenesis imperfecta and Ehlers-Danlos syndrome. *J. Bone Miner. Res.* **29**, 1412–1423. 10.1002/jbmr.2177 (2014). 24443344 10.1002/jbmr.2177

[18] Superti-Furga, A., Pistone, F., Romano, C. & Steinmann, B. Clinical variability of osteogenesis imperfecta linked to COL1A2 and associated with a structural defect in the type I collagen molecule. *J. Med. Genet.* **26**, 358–362. 10.1136/jmg.26.6.358 (1989). 2567784 10.1136/jmg.26.6.358 PMC1015618

[19] Smart, N. *et al.* Thymosin beta4 induces adult epicardial progenitor mobilization and neovascularization. *Nature* **445**, 177–182. 10.1038/nature05383 (2007). 17108969 10.1038/nature05383

[20] Le Cao, K. A., Rossouw, D., Robert-Granie, C. & Besse, P. A sparse PLS for variable selection when integrating omics data. *Stat. Appl. Genet. Mol. Biol.* **7**, Article 35. 10.2202/1544-6115.1390 (2008). 19049491 10.2202/1544-6115.1390

[21] Kitzler, T. M. *et al.* COL4A1 mutations as a potential novel cause of autosomal dominant CAKUT in humans. *Hum. Genet.* **138**, 1105–1115. 10.1007/s00439-019-02042-4 (2019). 31230195 10.1007/s00439-019-02042-4 PMC6745245

[22] *Hippo Signaling Pathway*. https://www.sciencedirect.com/topics/biochemistry-genetics-and-molecular-biology/hippo-signaling-pathway (2022).

[23] Wong, J. S., Meliambro, K., Ray, J. & Campbell, K. N. Hippo signaling in the kidney: The good and the bad. *Am. J. Physiol. Renal. Physiol.* **311**, F241-248. 10.1152/ajprenal.00500.2015 (2016). 27194720 10.1152/ajprenal.00500.2015 PMC5005280

[24] Sahay, M. Congenital anomalies of kidney and urinary tract (CAKUT). *Clin. Queries Nephrol.* **2**, 154–165. 10.1016/j.cqn.2013.11.005 (2013). 10.1016/j.cqn.2013.11.005

[25] Bierzynska, A., Soderquest, K. & Koziell, A. Genes and podocytes—New insights into mechanisms of podocytopathy. *Front. Endocrinol. (Lausanne)* **5**, 226. 10.3389/fendo.2014.00226 (2014). 25667580 10.3389/fendo.2014.00226 PMC4304234

[26] Kuo, D. S., Labelle-Dumais, C. & Gould, D. B. COL4A1 and COL4A2 mutations and disease: Insights into pathogenic mechanisms and potential therapeutic targets. *Hum. Mol. Genet.* **21**, R97-110. 10.1093/hmg/dds346 (2012). 22914737 10.1093/hmg/dds346 PMC3459649

[27] Pohl, M., Stuart, R. O., Sakurai, H. & Nigam, S. K. Branching morphogenesis during kidney development. *Annu. Rev. Physiol.* **62**, 595–620. 10.1146/annurev.physiol.62.1.595 (2000). 10845104 10.1146/annurev.physiol.62.1.595

[28] Jovanovic, I. *et al.* Transcriptome-driven integrative exploration of functional state of ureter tissue affected by CAKUT. *Life Sci.* **212**, 1–8. 10.1016/j.lfs.2018.09.042 (2018). 30261159 10.1016/j.lfs.2018.09.042

[29] Mitrovic, K. *et al.* A preliminary study of the miRNA restitution effect on CNV-induced miRNA downregulation in CAKUT. *BMC Genom.* **25**, 218. 10.1186/s12864-024-10121-8 (2024). 10.1186/s12864-024-10121-8 PMC10900603 38413914

[30] van der Ven, A. T., Vivante, A. & Hildebrandt, F. Novel insights into the pathogenesis of monogenic congenital anomalies of the kidney and urinary tract. *J. Am. Soc. Nephrol.* **29**, 36–50. 10.1681/ASN.2017050561 (2018). 29079659 10.1681/ASN.2017050561 PMC5748921

[31] Huang, H. Y. *et al.* miRTarBase 2020: Updates to the experimentally validated microRNA-target interaction database. *Nucleic Acids Res.* **48**, D148–D154. 10.1093/nar/gkz896 (2020). 31647101 10.1093/nar/gkz896 PMC7145596

[32] Fedou, C. *et al.* Mapping of the amniotic fluid proteome of fetuses with congenital anomalies of the kidney and urinary tract identifies plastin 3 as a protein involved in glomerular integrity. *J. Pathol.* **254**, 575–588. 10.1002/path.5703 (2021). 33987838 10.1002/path.5703

[33] da Silva, B., Santos, L. O., Burger, K., Kaliyaperumal, R. & Wilkinson, M. D. FAIR data point: A FAIR-oriented approach for metadata publication. *Data Intelligence.*10.1162/dint_a_00160 (2022). 10.1162/dint_a_00160

[34] Soiland-Reyes, S. *et al.* Packaging research artefacts with RO-crate. *Data Sci.* **5**, 97–138. 10.3233/DS-210053 (2022). 10.3233/DS-210053

[35] Singh, A. *et al.* DIABLO: An integrative approach for identifying key molecular drivers from multi-omics assays. *Bioinformatics* **35**, 3055–3062. 10.1093/bioinformatics/bty1054 (2019). 30657866 10.1093/bioinformatics/bty1054 PMC6735831

[36] Shen, R., Olshen, A. B. & Ladanyi, M. Integrative clustering of multiple genomic data types using a joint latent variable model with application to breast and lung cancer subtype analysis. *Bioinformatics* **25**, 2906–2912. 10.1093/bioinformatics/btp543 (2009). 19759197 10.1093/bioinformatics/btp543 PMC2800366

[37] Chalise, P. & Fridley, B. L. Integrative clustering of multi-level ’omic data based on non-negative matrix factorization algorithm. *PLoS One* **12**, e0176278. 10.1371/journal.pone.0176278 (2017). 28459819 10.1371/journal.pone.0176278 PMC5411077

[38] Lock, E. F., Hoadley, K. A., Marron, J. S. & Nobel, A. B. Joint and individual variation explained (Jive) for integrated analysis of multiple data types. *Ann. Appl. Stat.* **7**, 523–542. 10.1214/12-AOAS597 (2013). 23745156 10.1214/12-AOAS597 PMC3671601

[39] Bady, P., Doledec, S., Dumont, B. & Fruget, J. F. Multiple co-inertia analysis: A tool for assessing synchrony in the temporal variability of aquatic communities. *C. R. Biol.* **327**, 29–36. 10.1016/j.crvi.2003.10.007 (2004). 15015753 10.1016/j.crvi.2003.10.007

[40] Argelaguet, R. *et al.* Multi-Omics Factor Analysis-a framework for unsupervised integration of multi-omics data sets. *Mol. Syst. Biol.* **14**, e8124. 10.15252/msb.20178124 (2018). 29925568 10.15252/msb.20178124 PMC6010767

[41] Tenenhaus, M., Tenenhaus, A. & Groenen, P. J. F. Regularized generalized canonical correlation analysis: A framework for sequential multiblock component methods. *Psychometrika.*10.1007/s11336-017-9573-x (2017). 28536930 10.1007/s11336-017-9573-x

[42] Zitnik, M. & Zupan, B. Data fusion by matrix factorization. *IEEE Trans. Pattern Anal. Mach. Intell.* **37**, 41–53. 10.1109/TPAMI.2014.2343973 (2015). 26353207 10.1109/TPAMI.2014.2343973

[43] Teschendorff, A. E., Jing, H., Paul, D. S., Virta, J. & Nordhausen, K. Tensorial blind source separation for improved analysis of multi-omic data. *Genome Biol.* **19**, 76. 10.1186/s13059-018-1455-8 (2018). 29884221 10.1186/s13059-018-1455-8 PMC5994057

[44] UniProt, C. UniProt: The universal protein knowledgebase in 2021. *Nucleic Acids Res.* **49**, D480–D489. 10.1093/nar/gkaa1100 (2021). 33237286 10.1093/nar/gkaa1100 PMC7778908

[45] Raudvere, U. *et al.* g:Profiler: A web server for functional enrichment analysis and conversions of gene lists (2019 update). *Nucleic Acids Res.* **47**, W191–W198. 10.1093/nar/gkz369 (2019). 31066453 10.1093/nar/gkz369 PMC6602461

[46] Pan, M. & Zhang, J. Quantile normalization for combining gene-expression datasets. *Biotechnol. Biotechnol. Equip.* **32**, 751–758 (2018). 10.1080/13102818.2017.1419376

[47] Zhao, Y., Wong, L. & Goh, W. W. B. How to do quantile normalization correctly for gene expression data analyses. *Sci. Rep.* **10**, 15534. 10.1038/s41598-020-72664-6 (2020). 32968196 10.1038/s41598-020-72664-6 PMC7511327

