# Supplementary material for: Integrative analysis of multi-omics data reveals importance of collagen and the PI3K AKT signalling pathway in CAKUT
Source: Sci Rep. 2024 Sep 5;14:20731. doi: 10.1038/s41598-024-71721-8 (PMC11377713; doi:10.1038/s41598-024-71721-8)
Supplement: Supplementary file 6 — Supplementary Figure 1. [file 41598_2024_71721_MOESM6_ESM.docx]

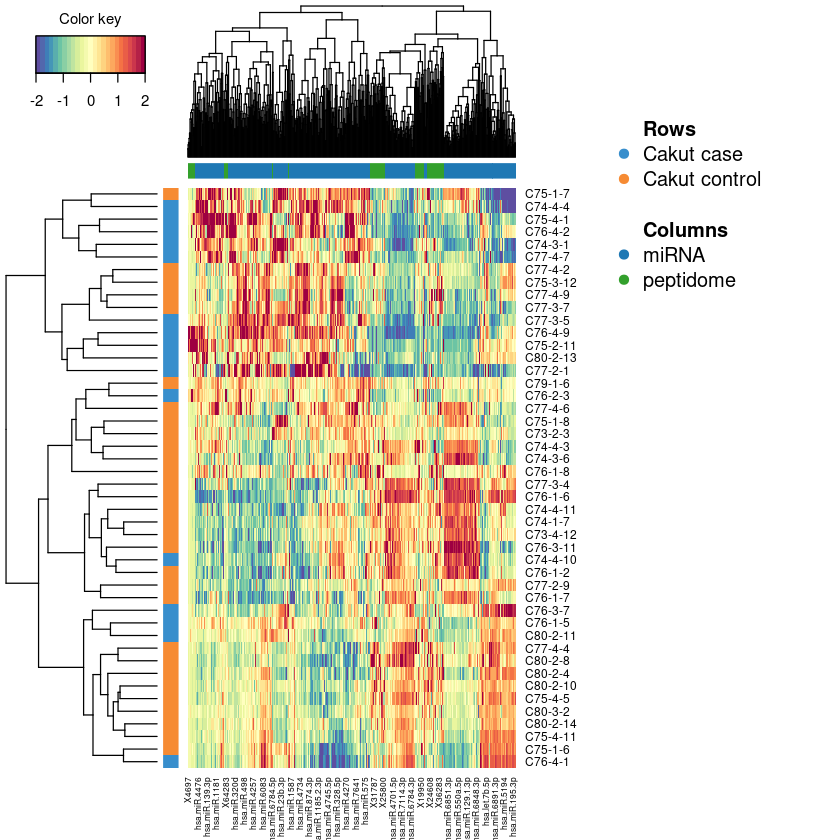


**Supplementary Figure 1:** Correlation plot between miRNA and peptidome. The table below lists the peptides and miRNAs from the figure in better readable way.

| Peptides | miRNAs |
| --- | --- |
| 64283  4697  36283  31787  25800  24608  19950 | hsa.miR.874.3p  hsa.miR.7641  hsa.miR.7114.3p  hsa.miR.6891.3p  hsa.miR.6851.3p  hsa.miR.6848.3p  hsa.miR.6784.5p  hsa.miR.6784.3p  hsa.miR.6083  hsa.miR.575  hsa.miR.550z.5p  hsa.miR.5194  hsa.miR.498  hsa.miR.4745.5p  hsa.miR.4734  hsa.miR.4701.5p  hsa.miR.4476  hsa.miR.4270  hsa.miR.4257  hsa.miR.328.5p  hsa.miR.320d  hsa.miR.23b.3p  hsa.miR.195.3p  hsa.miR.1587  hsa.miR.139.3p  hsa.miR.129.1.3p  hsa.miR.1185.2.3p  hsa.miR.1181  hsa.let.7b.5p |
